# In *vitro* nephrotoxicity and anticancer potency of newly synthesized cadmium complexes

**DOI:** 10.1038/s41598-019-51109-9

**Published:** 2019-10-11

**Authors:** Selda Abyar, Ali Akbar Khandar, Roya Salehi, Seyed Abolfazl Hosseini-Yazdi, Effat Alizadeh, Mehrdad Mahkam, Amer Jamalpoor, Jonathan M. White, Motahhareh Shojaei, O. Aizpurua-Olaizola, Rosalinde Masereeuw, Manoe J. Janssen

**Affiliations:** 1 Department of Inorganic Chemistry, Faculty of Chemistry, University of Tabriz, Tabriz, 5166614766 Iran; 2 Drug Applied Research Center and Department of Medical Nanotechnology, Faculty of Advanced Medical Science, Tabriz University of Medical Sciences, Tabriz, 51656–65811 Iran; 3 Chemistry Department, Faculty of Science, Azerbaijan Shahid Madani University, Tabriz, 5375171379 Iran; 4 Division of pharmacology, Utrecht Institute for Pharmaceutical Sciences, Utrecht University, Universiteitsweg 99, 3584 CG Utrecht, Netherlands; 5 School of Chemistry and BIO-21 Institute, University of Melbourne, Parkville, Vic. 3010 Australia; 6 Department of Chemical Biology and Drug Discovery, Utrecht Institute for Pharmaceutical Sciences, Utrecht University, Universiteitsweg 99, Utrecht, Netherlands

**Keywords:** Chemotherapy, Drug development, Toxicology

## Abstract

Complexes based on heavy metals have great potential for the treatment of a wide variety of cancers but their use is often limited due to toxic side effects. Here we describe the synthesis of two new cadmium complexes using N(4)-phenyl-2-formylpyridine thiosemicarbazone (L1) and 5-aminotetrazole (L2) as organic ligands and the evaluation of their anti-cancer and nephrotoxic potential in *vitro*. The complexes were characterized by Single-crystal X-ray data diffraction, ^1^HNMR, FT-IR, LC/MS spectrometry and CHN elemental analysis. Next, cytotoxicity of these cadmium complexes was evaluated in several cancer cell lines, including MCF-7 (breast), Caco-2 (colorectal) and cisplatin-resistant A549 (lung) cancer cell lines, as well as in conditionally-immortalized renal proximal tubule epithelial cell lines for evaluating nephrotoxicity compared to cisplatin. We found that both compounds were toxic to the cancer cell lines in a cell-cycle dependent manner and induced caspase-mediated apoptosis and caspase-independent cell death. Nephrotoxicity of these compounds was compared to cisplatin, a known nephrotoxic drug, *in vitro*. Our results demonstrate that compound {2}, but not compound {1}, exerts increased cytotoxicity in MCF-7 and A549 cell lines, combined with reduced nephrotoxic potential compared to cisplatin. Together these data make compound {2} a likely candidate for further development in cancer treatment.

## Introduction

Platinum-based complexes like cisplatin, oxaliplatin and carboplatin are used for the treatment of a wide variety of cancers^1^ including testicular, ovarian, bladder, breast cancer and melanoma^2^. Despite their wide use, platinum compounds have serious side effects including neurotoxicity, ototoxicity and dose-dependent nephrotoxicity^3^. In addition, these compounds are not useful in the treatment of platinum resistant cancers, such as non-small lung cancer and renal cell carcinoma. These problems have prompted chemists to synthesize anticancer compounds from other heavy metals including ruthenium, cobalt, gold iron and cadmium^4–8^. Cadmium is as an abundant metal in the environment, found in natural sources. However, its intake can lead to cadmium accumulation in the proximal tubules cells (PTC) of the kidney, potentially leading to renal dysfunction^9,10^. Pharmacological uses of this compound have therefore been limited. Recent studies showed the anticancer potential of several cadmium complexes but did not consider the nephrotoxicity of this metal^11–17^. The Cd (II) ion shows high affinity for soft donor atoms like sulfur and nitrogen (S ≫ N). In the human body cadmium will stimulate the synthesis of thiol rich metallothionein (MT) proteins, and form a Cd-MT complex to facilitate renal excretion^10^. Some studies showed that this complex has lower toxicity than the initial cadmium ion, indicating that metallothionein has a protective function by chelating the Cd(II) ion^10^. Also, chelating agents like ethylenediaminetetraacetic acid (EDTA) and thiacalixarenes can be used to bind Cd(II) and increase urinary excretion of this metal^18–20^. We therefore hypothesize that cadmium in a complex could be less nephrotoxic while remaining part of its anticancer properties. Previously, studies have also reported potential anticancer activity and considerable cellular membrane permeability of thiosemicarbazide derivatives for pharmaceutical applications, showing that theses organic ligands play an important role in drug delivery/distribution^21^. Here, we describe the synthesis and structural analysis of two new cadmium complexes. To study their anticancer activity, we used three cancer cell lines; MCF-7 (breast cancer), Caco-2 (colorectal cancer) and the cisplatin resistant A549 (lung cancer cell line). To evaluate the renal handling and nephrotoxic potential of the newly synthesized cadmium complexes, conditionally immortalized human proximal tubule epithelial cells (ciPTEC) were used. These cells have been thoroughly characterized and proven to be equipped with a broad range of transporter proteins, including organic anion transporter 1 (OAT1) and organic cation transporter 2 (OCT2) at the basolateral membrane and diverse ATP binding cassette (ABC) transporters at the luminal side, relevant for renal xenobiotic handling^22–26^. For comparison of the efficacy and safety profiles of the new cadmium complexes, cisplatin was used as a reference drug.

## Results

### Synthesis of two new cadmium complexes

**Figure 1:**
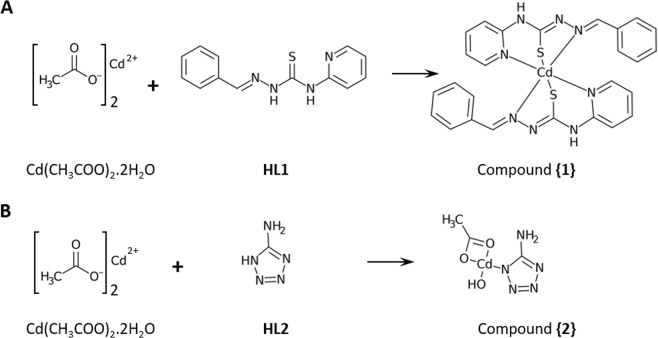
Synthesis of cadmium based compounds. (**A**) Compound {1} (Cd(L1)_2_.CH_3_OH) was synthesized by combining Cd(CH_3_COO)_2_.2H_2_O and N(4)-phenyl-2-formylpyridine thiosemicarbazone (HL1). (**B**) Compound {2} (Cd(L2)(CH_3_COO)(H_2_O)) was formed from an aqueous solutions of Cd(CH_3_COO)_2_.2H_2_O and 5-aminotetrazole (HL2).

In this study, firstly, we used a Schiff base chelating agent containing sulfur and nitrogen groups, N (4)-phenyl-2-formylpyridine thiosemicarbazone (HL1) to chelate the Cd(II) ion. This polar ligand interacts with Cd(II), resulting in a new complex Cd(L1)_2_.CH_3_OH, complex {1} (Fig. 1A). Secondly, due to the ability of tetrazole group to decrease the toxicity of metals^27^, we used 5-aminotetrazole (HL2) as a coordinating agent. The nitrogen atoms in this compound can easily chelate the Cd(II) ion and produce (Cd(aminotetrazole) (CH_3_COO) (H_2_O), complex {2} (Fig. 1B).

### Cytotoxicity of compounds {1} and {2} in cancer and kidney cells

**Figure 2:**
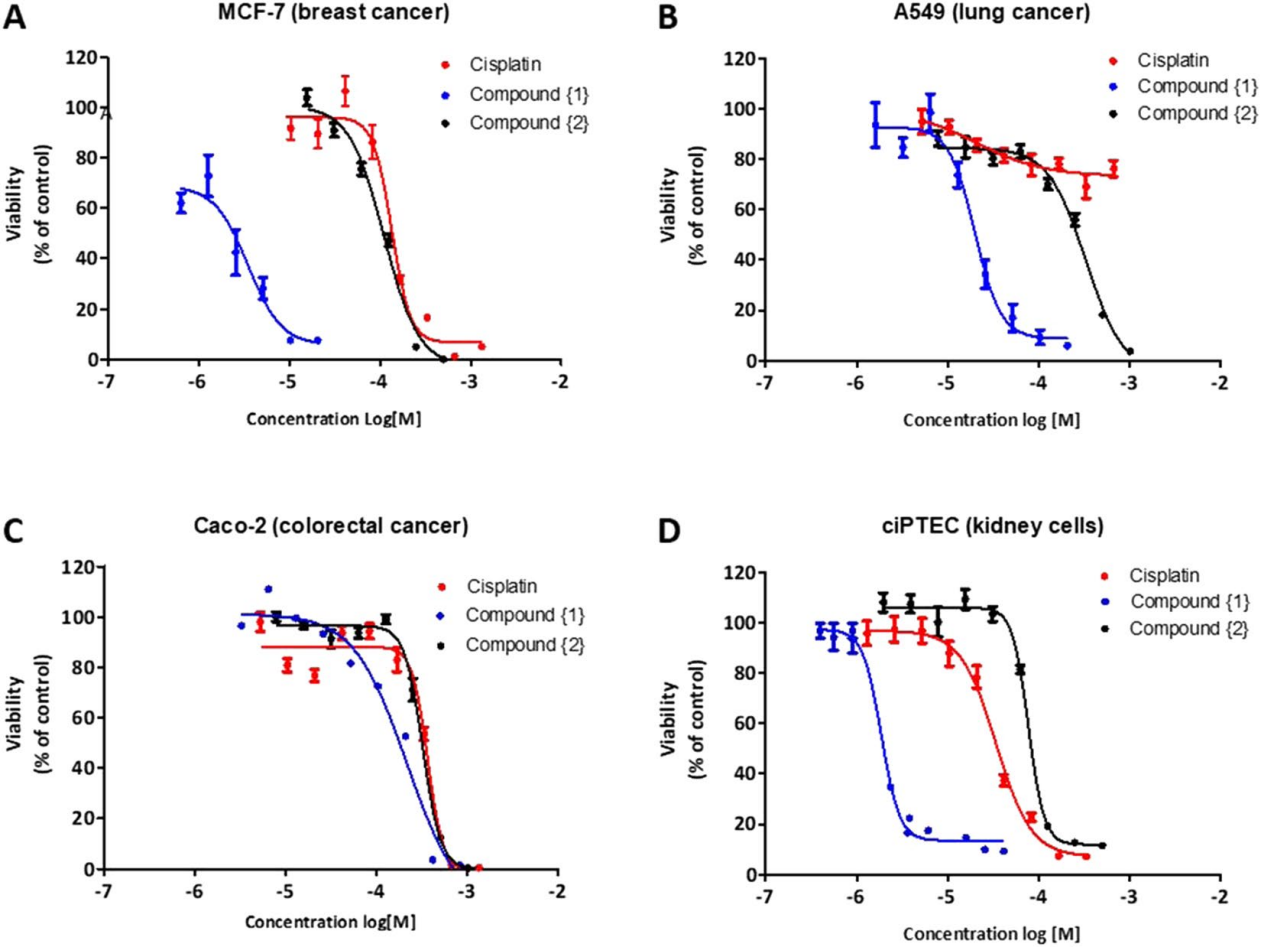
Cytotoxicity of cadmium compounds. Three different cancer cell lines (**A**-**C**) and one kidney cell line (**D**) were exposed for 24 h or 48 h to different concentrations of cisplatin (red line), compound {1} (blue line) or compound {2} (black line). Cell viability was evaluated with Presto- Blue and is shown relative to untreated cells (set to 100%).

To evaluate the potency of complex {1} and complex {2} as anti-cancer drugs, we exposed three different cancer cell lines to increasing concentrations of compound {1} or {2} and determined their TC_50_ values (Fig. 2 and Table 1). The Caco-2 cells were very resistant to all compounds and we had to extended the exposure time to 48 h before evaluating their cytotoxicity (Fig. 2C). As the Caco-2 cells were not sensitive to the compounds and we did not see any differences between the compounds we did not include them in the follow-up experiments. Both cadmium compounds showed cytotoxicity towards the three cancer cell lines (Fig. 2A–C), similar or at even lower concentrations than the reference drug cisplatin. However, the two compounds showed very different TC_50_ values. Compound {1} was about 30 fold more toxic to MCF-7 cells compared to compound {2} and cisplatin, and 15 times more toxic than compound {2} in lung cancer cells. As expected, cisplatin did not show any cytotoxicity (up to 1 mM) in the cisplatin resistant lung cancer cell line (Fig. 2B).

**Table 1:** Cytotoxicity evaluation.

	TC_50_ (µM)		
	Cisplatin	Compound {1}	Compound {2}
MCF-7	97 ± 2	3.00 ± 0.01	83 ± 2
Caco-2 (After 48 h)	283 ± 2	81 ± 2	267 ± 2
A549	>1000	14.0 ± 1.1	220 ± 3
ciPTEC14.4	30 ± 1	2.00 ± 0.01	78 ± 1

Data are expressed as mean ± SEM (n = 3–7).

Next, we tested the toxic effect of the compounds on kidney cells in comparison to cisplatin. Compound {1}, with the higher cytotoxic potency (p < 0.01) for all cancer cell lines studied, was also more toxic for ciPTEC (Fig. 2D). In contrast, compound {2}, while displaying a better cytotoxicity than cisplatin in the cancer cell lines, showed lower nephrotoxicity in ciPTEC (p < 0.01) (Fig. 2D). An overview of all cytotoxicity data is presented in Table 1.

### Compound {1} and {2} induce apoptosis and arrest cells in G2/M phase

**Figure 3:**
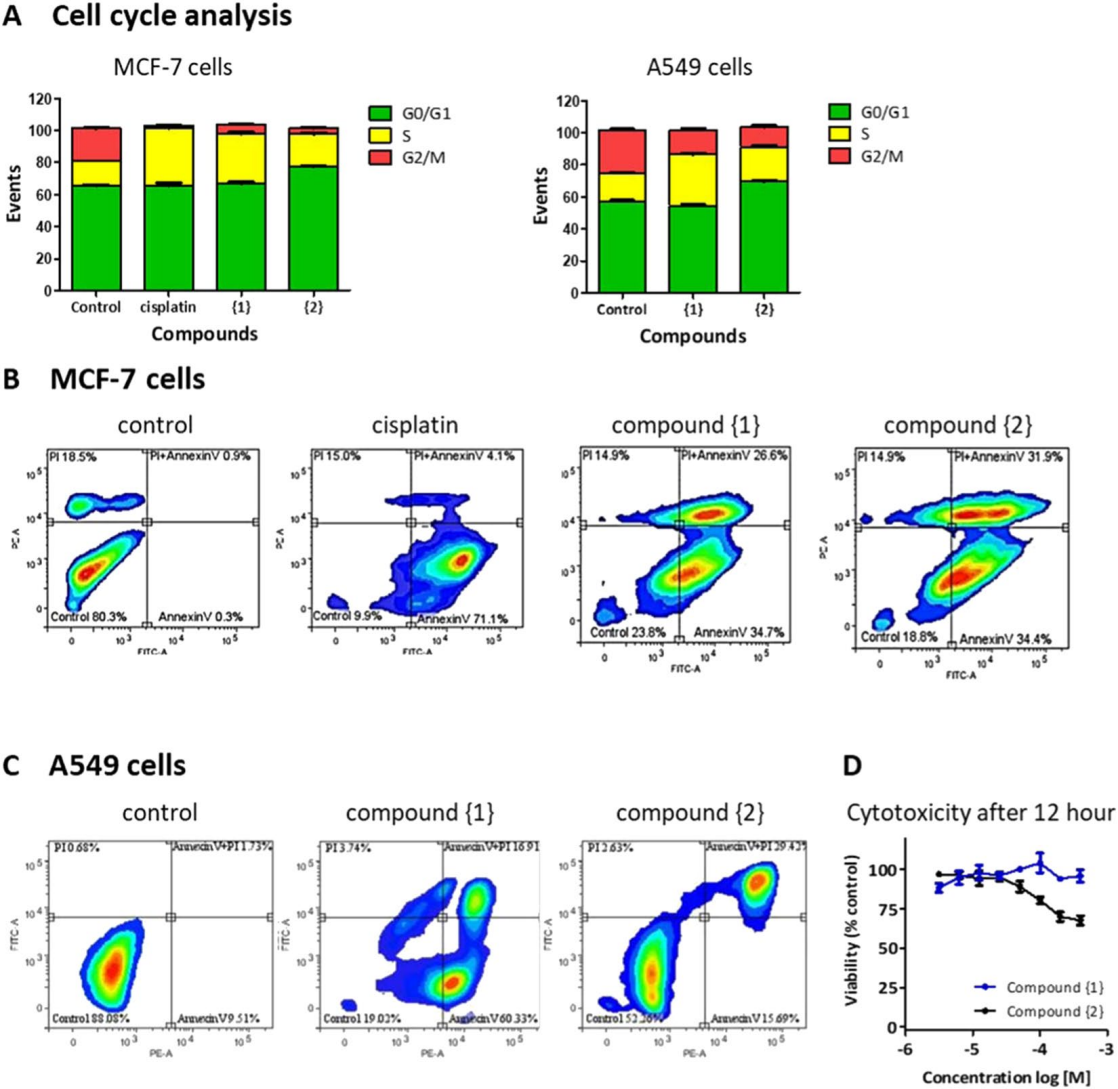
Cell cycle arrest and apoptosis. MCF-7 and A549 cells were incubated with compound {1} and {2} (and cisplatin in case of MCF-7) at their TC_50_ for 24 hr. (**A**) Cell cycle analysis was performed by staining the DNA content of the cell followed by flow cytometry, percentage of cells in G0/G1, S or G2/M phase is indicated. Annexin V and PI staining (**B**,**C**) was used to identify viable cells (annexin V−, PI−), early apoptotic cells (annexin V+, PI−), late apoptotic or necrotic cells (annexin V+, PI+) and necrotic cells (annexin V−, PI+). Cytotoxicity of compounds {1} and {2} was also evaluated after 12 h in A549 cells (D). Control cells were cultured in the absence of compound but in the presence of 0.5% DMSO (vehicle).

Anticancer drugs can be classified as cell cycle specific (CCSS) or cycle non–specific (CCNS). Cisplatin is known to be cell cycle specific, arresting cells in S and G2/M phase due to binding of cisplatin to DNA, causing DNA damage^2,28–30^. Here we evaluated the percentage of cells in G0/G1, S or G2/M phase under normal growth conditions (control) and after exposure to either cisplatin, compound {1} or compound {2} (Fig. 3A). In the MCF-7 cells, exposure to cisplatin indeed resulted in a cell cycle arrest in the S-phase which blocked the progression to G2/M phase. As the A549 cells are cisplatin resistant (see also Fig. 2B), we could not establish a TC_50_ value or evaluate the effect of cisplatin on cell cycle in the A549 cells. Compounds {1} and {2}, like cisplatin, reduced the number of cells in G2/M phase of MCF-7 and A549 cells (Fig. 3A), thereby acting as cell-cycle specific chemotherapeutic agents *in vitro*.

Annexin V is a fluorescent probe that binds to translocated phosphatidylserine in apoptotic cells and is used to indicate apoptotic cells, whereas PI stains the nucleus of necrotic or late apoptotic cells^31^. The Annexin V/PI staining showed that in MCF-7 cells treated with cisplatin the majority of cells (71.1%) were in early apoptosis within 24 h, whereas in the presence of cadmium compounds both early and late apoptotic cells (respectively 35% and 27% for compound {1}, 34% and 32% for compound {2}) were found at this time (Fig. 3B). This shows that in the presence of cadmium compounds, the complete apoptosis process took place within 24 h. As shown in Fig. 3C, in A549 cells in presence of {2}, only late apoptotic cells were observed, but in the presence of {1}, both early and late apoptotic cells were visible. This suggests that in this cell line, compound {2} kills the cells faster than compound {1}. The cytotoxicity of the two cadmium compounds was therefore evaluated after 12 h as well (Fig. 3D) and indeed {2} showed more cytotoxicity than {1} during this short-term exposure.

### Activation of caspase dependent and independent apoptosis pathways in cancer cells

**Figure 4:**
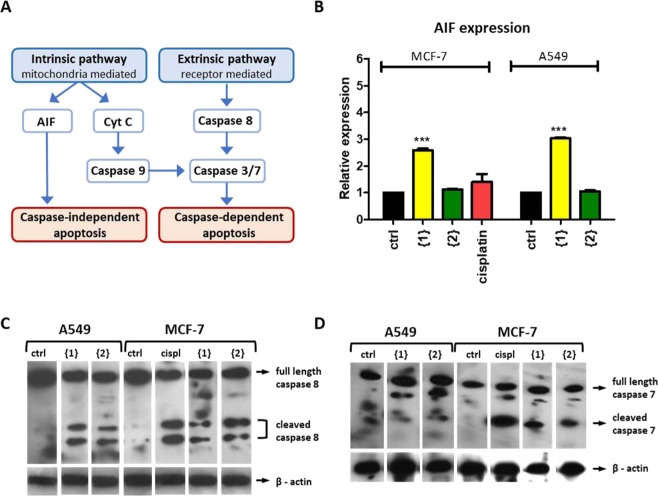
Pathways involved in apoptosis. (**A**) Schematic overview of factors involved in intrinsic and extrinsic apoptosis pathway. (**B**) quantitative PCR of AIF in MCF-7 and A549 cells (normalized to GAPDH and relative to ctrl) (n = 3, ***p < 0.001). Western blot analysis of caspase 8 (**C**) and caspase 7 (**D**) activation. Ctrl: untreated cells, {1}: compound {1}, {2}: compound {2}, cispl: cisplatin.

Next we evaluated the role of caspase-dependent and caspase-independent programmed cell death pathways (Fig. 4A). As expected, cisplatin treatment showed a strong activation of the (caspase dependent) intrinsic pathway in MCF-7 cells (Fig. 4C,D), but did not result in expression of apoptosis inducing factor (AIF) which is specific for caspase independent apoptosis (Fig. 4B).

Treatment with compound {1} and {2} resulted in activation of caspase 8 in both MCF-7 and A549 cells, and increased levels of activated effector caspase 7 in MCF-7 (Fig. 4C,D). However, levels of caspase 7 did not seem to increase in the cisplatin resistant A549 cancer cells, another executioner caspase may be responsible for apoptosis in A549 cells. Remarkably, compound {1} did show a clear increase in the expression of AIF in both A549 and MCF-7 cells (p < 0.001) (Fig. 4B), indicating this compound targets both the caspase-dependent and caspase-independent apoptosis pathways.

### Compound {2} displays good chemotherapeutic potential with reduced nephrotoxicity compared to cisplatin

**Figure 5:**
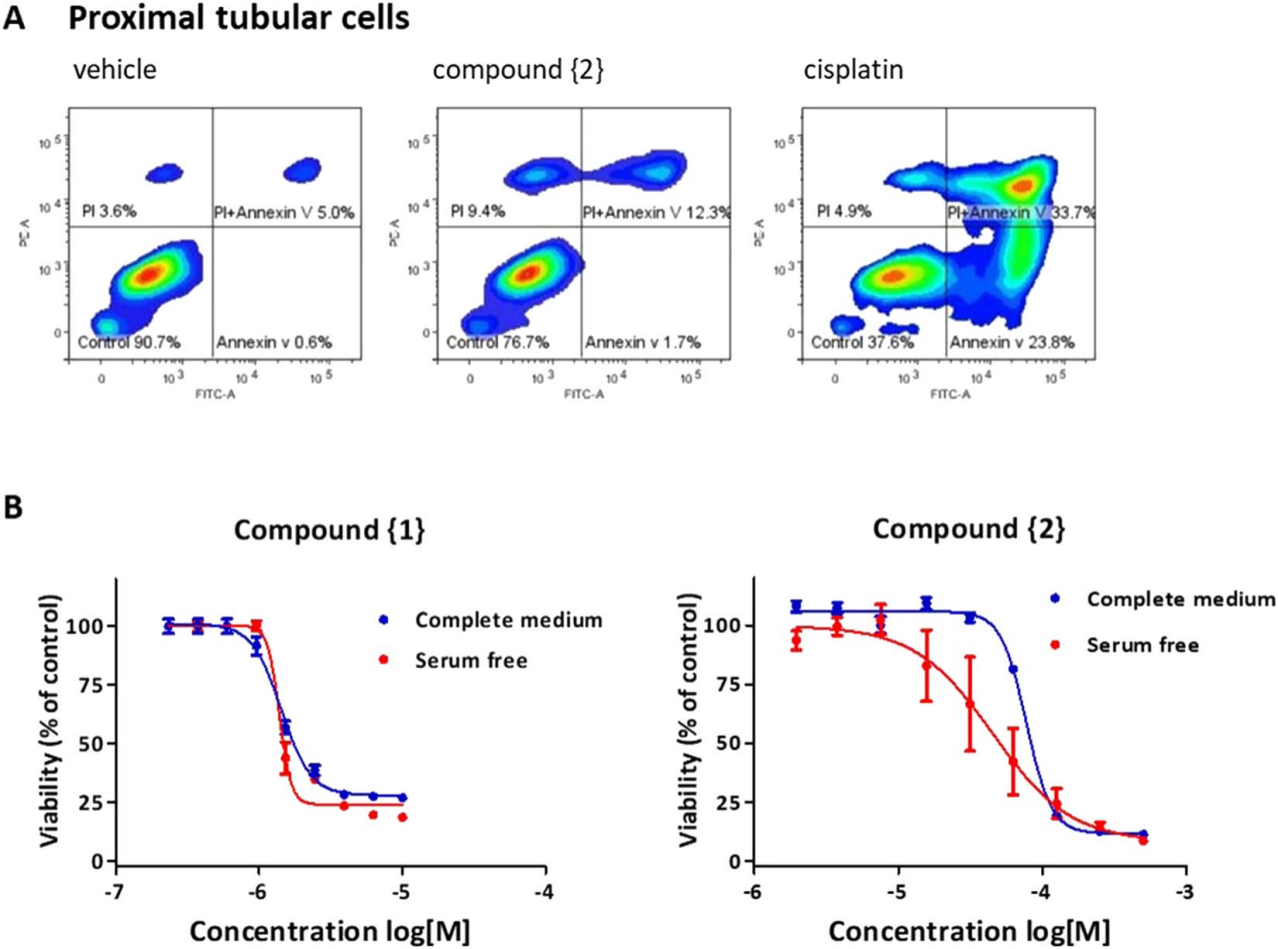
Cytotoxicity in kidney cells. (**A**) Exposure of ciPTEC with 50 µM of either compound {2} or cisplatin for 24 h shows higher sensitivity to cisplatin evaluated by Annexin V/PI staining using flow cytometry. (**B**) Cytotoxicity of compound {1} and {2} in ciPTEC the presence or absence of serum.

The main dose-limiting side effect of cisplatin is nephrotoxicity. The cell viability assay (Fig. 2) used to determine the TC_50_ (Table 1) of cisplatin and the cadmium complexes showed that compound {2} had lower nephrotoxic potential compared to cisplatin while having a comparable or more potent effect than cisplatin on the cancer cells. To confirm the reduced sensitivity in kidney cells, we evaluated the Annexin V/PI staining in ciPTEC after exposure to the same concentration (50 µM) of either compound {2} or cisplatin for 24 h. Based on the cell viability assay (Fig. 2) 50 µM is toxic to both cell lines and lays between the IC50 values of these two cell lines. As shown in Fig. 5A, in the presence of compound {2} only a small increase in apoptotic cells was seen compared to control, whereas in the presence of the same concentration of cisplatin most cells were apoptotic. This confirms that {2} exhibits lower nephrotoxic potential than cisplatin in ciPTEC cells.

### Effect of serum on toxicity of cadmium compounds in ciPTEC

Fetal calf serum (FCS) is a supplement used in many cell culture media for successful culturing. It contains not only growth factors and cytokines necessary for cell division, but also plasma proteins such as albumin. Many compounds can bind to these proteins in serum which may affect their uptake by the cells. Addition of FCS to cell culture medium can therefore cause an immediate change in transport rate. To assess the effect of FCS on the cytotoxicity of our cadmium compounds in ciPTEC, cells were treated with the cadmium complexes in both serum-free medium (SFM) or complete medium (containing 10% FCS). The Presto- Blue assay was used to study cell viability. The presence of serum did not affect the cytotoxicity of {1}, but the cytotoxicity of {2} was found to be increased in the absence of serum (Fig. 5B). This may indicate that a fraction of {2} is bound to plasma proteins and that the unbound fraction is slightly more toxic.

## Discussion

Cisplatin and other platinum compounds have had a huge impact in the treatment of cancers indicating the high potential of metal-based anticancer drugs. The goal of this study was to synthesize new cadmium-based complexes and evaluate their cytotoxic and nephrotoxic potential *in vitro*. Because of the high affinity of cadmium for nitrogen and sulfur atoms, we used organic compounds with nitrogen and sulfur (N (4)-phenyl-2-formylpyridine thiosemicarbazone (HL1)) or just nitrogen (5-aminotetrazole (HL2) as donor atoms in their structures. We successfully synthesized two new complexes, {1} and {2} (Fig. 1), and observed that these complexes showed comparable or higher cytotoxic potential in all cancer cell lines tested (Fig. 2 and Table 1). Furthermore, both compound {1} and {2} were toxic to the cisplatin resistant lung cancer cell line (A549) indicating they may be effective against cisplatin resistant tumors. Moreover, the cadmium complexes could induce cell death through apoptosis and arrest the cells in the S-Phase, acting as cell cycle-specific chemotherapeutic agent (CCSS) with potential genotoxicity *in vitro* (Fig. 3)^32^.

Nephrotoxicity is the dose limiting factor for cisplatin use due to its accumulation in the proximal tubule cells. Within the kidneys proximal tubule cells are responsible for the absorption and reabsorption of solutes and the excretion of xenobiotics, making them susceptible to drug-induced injury. Here we use human proximal tubular epithelial cells (ciPTEC) to evaluate the nephrotoxic potential of compound {1} and {2} *in vitro*^33^. We found that compound {2} had a lower toxicity in kidney cells compared to cisplatin and to be more potent in killing the cancer cells *in vitro*, therefore this compound looks very promising as a potential new anticancer drug. Furthermore, although compound {1} was much more toxic for kidney cells than cisplatin, this compound was even more potent in killing the cancer cells. Taking this into account the therapeutic window for both compound {1} and {2} may exceed that of cisplatin. The next step will be to test the efficacy and specificity of these compounds *in vivo* to determine the use of these compounds for anti-cancer therapy.

The structure and chemical properties of compound {1} and compound {2} are very different and will affect the way they are taken up and metabolized inside the cells. Depending on the chemical properties a compound can passively diffuse over the cell membrane, pass the cell membrane through specific transport proteins or channels, or enter the cell through receptor mediated endocytosis. The way compounds enter the cell can also affect their metabolism, their toxicity and elimination route. Based on its size and lipophilicity compound {1} is expected to easily pass the cell membrane by diffusion. This may explain why compound {1} was toxic at a much lower concentration than cisplatin or compound {2} in all cells tested (Table 1). Once inside the cell compound {1} may be metabolized into a more hydrophilic compound, leading to its accumulation inside the cells. Some drug compounds are highly protein-bound in the blood, effectively lowering their free concentration and toxic effects in the presence of proteins. For some compounds, however, receptor mediated endocytosis is the primary mode of entry into the cell and the absence of serum proteins may prevent their uptake into the cell. We found that the presence of serum proteins did not effect the toxicity profile of compound {1} in proximal tubule cells, but made the cells more sensitive to compound {2} (Fig. 5B). This suggests that both compound {1} and {2} do not require binding to serum proteins (like albumin) to be transported into the cells, but that a fraction of compound {2} may be protein bound.

Both compound {1} and {2} could induce apoptosis in a caspase dependent way, compound {1} also activated the caspase independent pathway in MCF7 and A549 cells. This means compound {1} may be able to modulate apoptosis in cells expressing caspase inhibitors such as XIAP, CrmA or p35^34,35^. We also found that compound {2} was able to induce apoptosis faster in A549 cells as seen by the large amount of late apoptotic cells after 24 h (Fig. 3D), and reduced cell viability within 12 h (Fig. 3D). This may be related to how the compound is metabolized inside the cell or the cellular pathways that are being targeted. We don’t know if the complexes themselves are toxic or that they are degraded and the cadmium is released inside the cells. Free cadmium is known to affect several processes in cells, including cell proliferation, differentiation, apoptosis, DNA repair and the production of reactive oxygen species (ROS)^9,36^. Chronic exposure can lead to genomic instability and tumorgenicity, so an important requirement for the therapeutic use of cadmium compounds is that their use does not lead to systemic accumulation of cadmium in patients.

In conclusion, the cadmium complexes described here may be interesting candidates for the development of a new class of anti-cancer drugs. Future studies should focus on addressing their efficacy against (cisplatin resistant) tumors, their specificity, safety and pharmacokinetic properties *in vivo*.

## Materials and Methods

Cd(CH_3_COO)_2_.2H_2_O and 5-aminotetrazole, cisplatin (cis-diamminedichloroplatinum(II)) were obtained from Sigma Aldrich, CA, USA. Culturing medium DMEM^TM^ and phenol red free medium DMEM/F12, PBS, FBS, FCS, Trypsin, Accutase enzyme, Penicillin, Presto- Blue^27^, Annexin V, propidium iodide (PI) solutions, RNA isolation and cDNA synthesis kits, primers, antibodies and Western blot reagents were obtained from Thermo Fisher Scientific and Gibco/Invitrogen, Breda, the Netherlands.

FT-IR spectra were determined on a Bruker Tensor 27 FT-IR spectrophotometer with KBr disks in the range of 4000–400 cm^−1^ (Columbia, MD, USA). QTOF LC/MS spectrometer was used for mass spectrometry (Billerica, MA, USA). ^1^HNMR spectroscopy were performed at room temperature on the NMR Bruker Advance 400 MHz magnet (Agilent, USA) using DMSO-*d*_6_. Elemental analysis, C H N, was performed by Elemental analyzer Vario EL III (Sydney, Australia). X- ray crystallography was performed on an SuperNova, Dual, Cu at zero, Atlas diffractometer, using Cu Kα radiation λ = 1.54184 Å at 130 K. Facs Canto II (Bio Rad) flow cytometry system (Mexico, USA) with Flow Logic TM program, was used for cell cycle AnnexinV and PI evaluation. Quantitative PCR (qPCR) was performed by Mic qPCR, Bio Molecular system (Santa Clara, USA).

### Synthesis and structural characterization of Cd(L1)_2_.CH_3_OH, compound {1}

All reactions were carried out with fresh distilled solvents. The (HL1) ligand, was prepared according to literature protocol^37^. Cadmium thiosemicarbazide {1} was synthesized by adding a methanol solution of HL1 (128 mg, 0.5 mmol) to methanol solution of Cd(CH_3_COO)_2_.2H_2_O (133.26 mg, 0.5 mmol). The reaction was refluxed for 4 h at 60 °C until the color of the solution turned to yellow. The mixture was cooled down to room temperature and after slowly evaporation of the solvent over 3 days, the yellow crystals precipitated.

**Figure 6:**
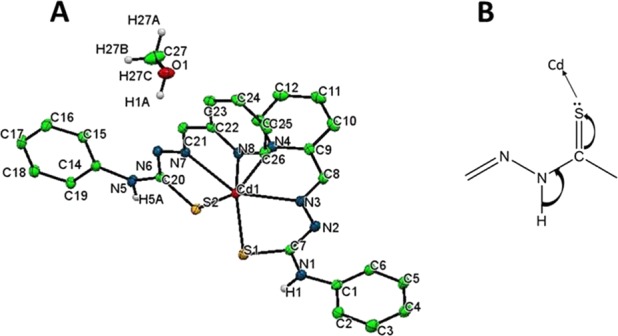
Structural Characterization of compound {1}. (**A**) ORTEP diagram of compound {1} and (**B**) deprotonation of hydrogen atom in the complexation process of HL1.

Further structural analysis was carried out by X-ray crystallography (crystallographic data ‘CCDC 1846149’ is deposited at the Cambridge Crystallographic Data Centre and can be accessed via www.ccdc.cam.ac.uk/data_request/cif). Crystal data were listed in Table 2 and ORTEP diagram was showed in Fig. 6A. In this complex, the cadmium(II) ion is coordinated by two thiosemicarbazide ligands (L1^−^) in a distorted octahedral environment. The lengths of both carbon-sulfur bonds in this complex (C7-S1: 1.7405(11) Å, C20-S2:1.7434(11) Å) show an increase in comparison to the carbon-sulfur bond in the free ligand (1.677(2) Å)^38^. The bond lengths of C7-N2 (1.316(2) Å) and C20-N6 (1.322(2) Å) in the complex decreased, compared to the free ligand (1.3685(2) Å)^38^. The shortening of mentioned C-N bonds and lengthening of C-S bonds in the complex compared to the corresponding bonds in the free ligand indicate that complexation occurs by losing the hydrogen atom attached to corresponding N6 or N2 atom of free ligand as shown in Fig. 6B.

**Table 2:** Crystal data and structure refinement parameters of compound {1}.

Chemical Formula	C27 H26 Cd N8 OS2
Formula weight	655.08
Crystal system	Monoclinic
Space group	P21/c
**Unit cell dimensions**	
a/ Å	13.3024 (1)
b/ Å	13.1676(2)
c/ Å	9 15.6750(2)
β/°	94.259 (1)
V/ Å3	2738.06
Z	4
T/K	130(10)
Wavelength	0.71073
Density (Calculated)	1.5899 gcm-1
µ(mm-1)	0.988
Index ranges/°h/k/l	−24 ≤ h ≤ 21
	−24 ≤ k ≤ 15
	−28 ≤ l ≤ 28
F (000)	1328
R int R1 (I > 2σ (I))	0.031
wR2 (Reflection)	0.0734(17569)
Goodness of fit (GOF) on F2	1.088

### Synthesis and characterization of Cd(L2)(CH_3_COO)(H_2_O), compound {2}

To synthesize compound {2}, 30 ml aqueous solution of Cd(CH_3_COO)_2_.2H_2_O (133.26 mg, 0.5 mmol) was added to 50 ml aqueous solution of 5-aminotetrazole (45.5 mg, 0.5 mmol). The mixture was stirred for 2 h and water slowly evaporated at the room temperature. Finally, the white precipitation collected and washed three times with water and dried at room temperature. This compound was characterized by FT-IR, liquid chromatography coupled to mass-spectrometry (LC/MS), ^1^HNMR and elemental analysis. Unfortunately, all attempts to prepare single crystals of this compound were unsuccessful.

The FT-IR spectrum of free ligand (HL2) shows symmetric and asymmetric (NH_2_) vibration bands observed in the 3382 cm^−1^ and 3486 cm^−1^, respectively The strong band at 3198 cm^−1^ in the spectrum of the free ligand assignable to (N-H) group disappeared from IR spectrum of complex suggesting the coordination of the ligand is accompanied by deprotonation of N4 hydrogen atom. This is also confirmed by ^1^HNMR of the complex. The appearance of a strong band at 1544 cm^−1^ in the complex spectrum is assignable to chelating carboxylate of acetate ion in the complex.

**Figure 7:**
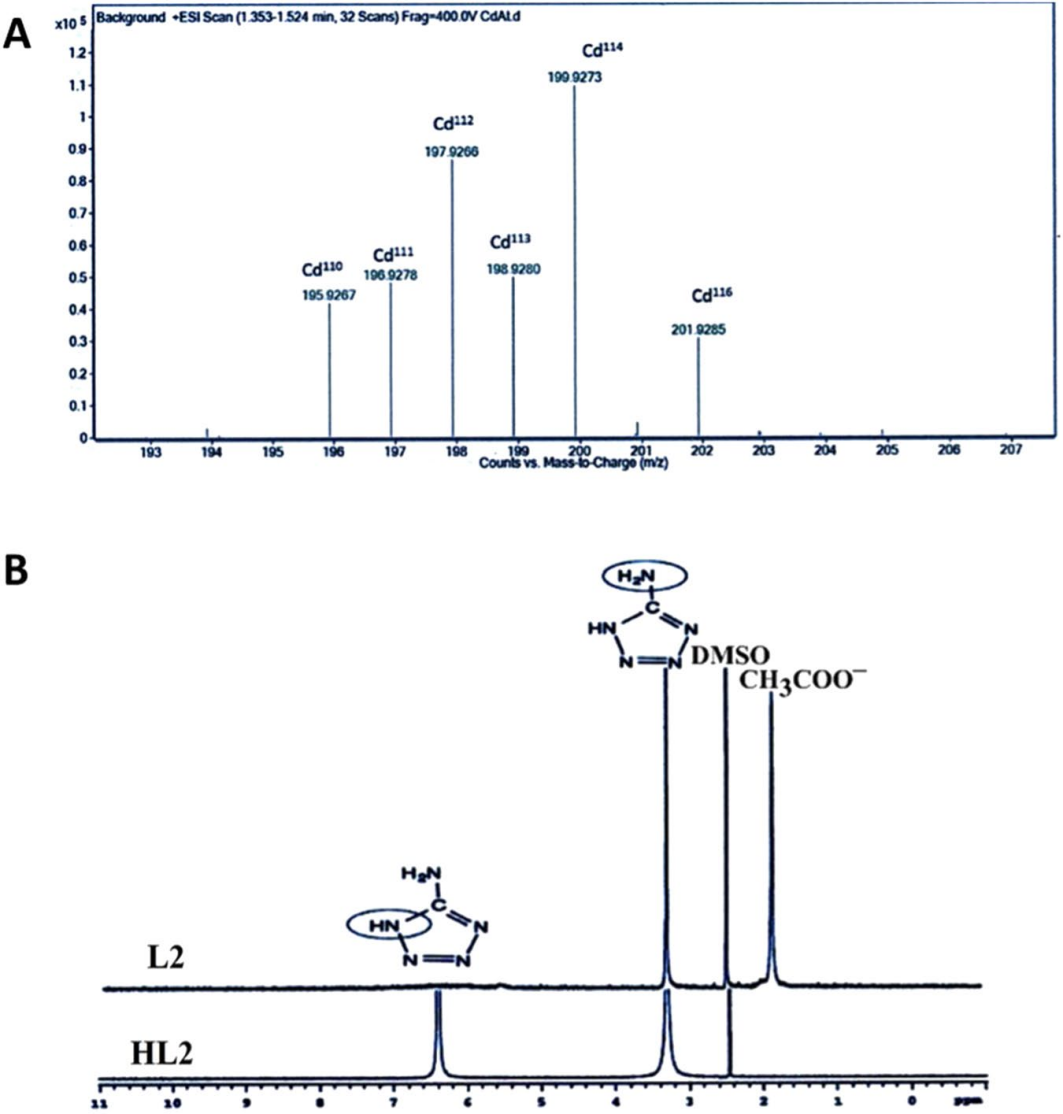
Structural characterization of compound {2}. (**A**) LC/MS spectrum of compound {2}, (**B**) ^1^HNMR of compounds, HL2 and {2}.

LC/MS spectrum of complex {2} (Fig. 7A) obtained from [ML+] by losing its acetate fragment fits with isotopic distribution of one cadmium and one aminotetrazole in this fragment and the most abundant experimental mass for [ML+] is m/z = 199.9273. In the ^1^HNMR spectrum of 5- aminotetrazole two bands appeared at 6.4 ppm and 3.3 ppm are related to (N-H) and NH_2_ groups, respectively. The (N-H) group band disappeared in the ^1^HNMR spectrum of complex {2} suggesting coordination of the ligand in the anionic form (L2^−^) to the metal center. Also, one new band appeared at 1.8 ppm in the spectrum of complex {2} is related to methyl group of acetate (Fig. 7B). Calculated mass for Cd(L2) (OAC) (H_2_O) is 273.531 and analytical data for this compound (%): C 13.17, H 2.58, N 25.60, Found (%): C 13.80, H 2.30, N 25.20. Therefore, based on all data we propose a four-coordinated geometry around Cd(II) surrounded by one deprotonated 5-aminotetrazole, one chelating acetate, and one water molecule. Our proposed structure for compound {2} is shown in Fig. 1B.

### Cell lines and cell culture

Human cancer cell lines, MCF-7, Caco-2 and A549, were obtained from American Type Culture Collection (Manassas, USA). The conditionally immortalized proximal tubule epithelial cells (ciPTEC) used in this study are described in detail elsewhere^23–25^. In brief, primary renal cells isolated from the urine of a healthy volunteer were immortalised by transduction with both human telomerase (hTERT) and the temperature sensitive SV40 large T antigen (SV40T). This was done to promote genome stability and cell expansion at 33 °C. A stable clone exhibiting robust proximal tubule characteristics was selected and characterised. Generally, all cell lines were grown as a monolayer. Cancer cells were seeded at an initial density of 48000 cells/cm^2^ and allowed to expand to reach the right density (which took approximately 3 days for MCF-7 and 7 days for A549) or form a confluent monolayer (21 days for Caco-2 cells) in Dulbecco’s Modified Eagle Medium, DMEM^TM^ with 10% fetal bovine serum (FBS and L-Glutamine and 100 IU/ml penicillin, at 37 °C in a 5% CO_2_ humidified atmosphere. ciPTEC were cultured in Dulbecco’s Modified Eagle Medium/Nutrient Mixture F-12 (1:1 DMEM/F-12) supplemented with 10% fetal calf serum (FCS), 5 µg/mL insulin, 5 µg/mL transferrin, 5 µg/mL selenium, 35 ng/mL hydrocortisone, 10 ng/mL epidermal growth factor and 40 pg/mL tri-iodothyronine, creating complete culture medium, without addition of antibiotics. Cells were seeded 7 days before the experiment at 55,000 cells/cm^2^ and grown for 1 day at 33 °C, followed by 6 days at 37 °C to confluency. All cell lines were passaged using Accutase® solution and incubated in a humidified atmosphere containing 5% (v/v) CO_2_, and all experiments were performed between passage 30 and 40.

### Cell viability assay

Cytotoxicity of cadmium compounds and cisplatin for all cell lines was assessed by Presto- Blue-based cell viability assay. Briefly, cells were plated in 96-well plates (population of MCF-7, A549 and Caco-2 was 1 × 10^4^ cells/well and for ciPTEC-14,4 it was 55 × 10^3^) then treated with nine subsequent dilutions of the drugs in triplicate (concentration range: 3–1200 µg/ml). Based on the sensitivity of the cells to the compounds the range was adjusted to obtain a reliable estimation of the toxicity as reflected by TC_50_ values (the drug concentration leading to a 50% reduction in cell viability). All the cell lines were treated for 24 h, Caco-2 cell line was treated for 24 and 48 h. Cells were washed with PBS and incubated with 10% Presto- Blue at 37 °C (optimal time of incubation was 30 min for cancer lines and 1 hr for for ciPTEC). The absorbance of the converted dye was recorded by Jasco spectrophotometer (Excitation wavelength: 560 nm, emission wavelength: 590 nm). Collected data used for TC_50_ prediction by GraphPad Prism^TM^.

### Cell cycle analysis

Cell cycle analysis is a method that is generally carried out by flow cytometry to identify the percentage of time that cells spent in different phases of the cell cycle. Before analysis, the cells were treated with a high concentration of PI. Cell cycle distribution of two more sensitive cancer cells (MCF-7 and A549) were determined by flow cytometry. To this end, cells were seeded in 6-well plates with 5 × 10^5^/well cells and treated with a concentration at the TC_50_-value of compounds for 24 h. Compounds were dissolved in DMSO resulting in a final concentration of 0.5% DMSO in the culture medium, the control condition consisted of cells cultured in the absence of compounds but in also the presence of 0.5% DMSO (vehicle). Cells were harvested after 24 h. Trypsinized cells were washed and fixed with 70% ethanol and stored overnight at 4 °C. 400 µl PI staining solution, consisting of 10 mg/ml RNase and 1 mg/ml PI and bovine serum albumin (0.1%), was added to the cell suspension. Facs Canto II (Bio Rad) flow cytometry system, with Flow Logic TM program, was used for cell cycle evaluation.

### Annexin V and propidium iodide (PI) staining

Annexin V-FITC and PI staining (Annexin V/PI) was performed for assessing the percentage of dead cells by apoptosis pathway. Briefly, lung and breast cancer cells were treated with a concentration at the TC_50_-value of all drugs for 24 h. For ciPTEC cell line, 50 µM of both cisplatin and compound {2} were used and cells were exposed to these compounds for 24 h. Subsequently, cells were washed with PBS, harvested and resuspended in 100 µl binding buffer consisting of 5 μL Annexin V-FITC. After incubation for 10 minutes, cells were washed and suspended in 200 μL of 1 × Binding Buffer. PI staining solution was added. Cells were analyzed by FACS canto II (Bio Rad) flow cytometry system. Existence of equal amounts of necrotic cells in control cells and treated cells showed cells were killed by pipetting errors.

### Real time quantitative PCR (qPCR)

Expression of the pro-apoptotic gene AIF was evaluated by qPCR to evaluate the caspase- independent apoptosis pathway in breast and lung cancer cell lines in presence of cadmium compounds. MCF-7 and A549 cells were treated with compounds for 24 h at their respective TC_50_-values. The cells were trypsinized and RNA was isolated by RNA isolation kit (Thermo Fisher Scientific) according to manufacturer’s instruction. The amount of RNA for each sample was measured by name-drop spectrophotometer (Bibby Scientific Ltd, UK). Subsequently, cDNA was synthesized using a cDNA synthesis kit (Thermo Fisher Scientific). Table 3 presents the primer sets used. Relative expression levels were calculated using the ${2}^{-{\Delta \Delta }_{CT}}$ method where GAPDH was used as reference gene for normalization. q-PCR was performed by Mic qPCR, Bio Molecular system.

**Table 3:** Primer sequences and annealing temperatures.

Gene	Primer Sequence	Temperature
AIF	Forward: GATTGCAACAGGAGGTACTCCAAGA	59 °C
	Reverse: GATTTGACTTCCCGTGAAATCTTCTC	
GAPDH	Forward: TGCACCACCAACTGCTTAGC	61 °C
	Reverse: GGCATGGACTGTGGTCATGAG	

### Western blot

Harvested cells (10^6^) were homogenized in ice cold RIPA lysis buffer containing protease inhibitor cocktail (Sigma Aldrich, CA, USA). The supernatant was collected after centrifuge for 20 min at 12000 RPM and 4 °C. The protein concentration was quantified using the Bradford assay w1–8ith commercial reagents (Bio-Rad, Des Plaines, USA) and spectrophotometric measurements (Bibby Scientific Ltd, Beacon Rd, UK). Proteins were separated by SDS-PAGE (10 µg protein loaded per each well) and transferred to Polyvinylidene difluoride (PVDF) membrane. Blots were blocked for 2 h with blocking the buffer containing 5% (w/v) nonfat dry milk in 1 × TBS 1% Tween®20 (TBST), then incubated overnight with primary antibodies (Caspase-7 (C7); cell signaling (1:1000), Anti-caspase 8 antibody: (Abcam Cambridge, USA) 1:1250, and Anti-β-actin: (Santa Cruz, USA) 1:500 diluted in the blocking buffer. On the next day, after washing three times in TBST, the membranes were incubated for 1 h with appropriate secondary antibody diluted in the blocking buffer. Enhanced chemiluminescence detection kit (Thermo Fisher Scientific, Breda, the Netherlands) was used for the visualization of blots, measurements were done in an Amersham Imager 600 (GE Healthcare Life Sciences, Eindhoven, The Netherlands). Bands we quantified using Image J and β-actin was used as a loading control.

### Data Analysis

Graphpad prism^TM^ 5.03 was used for analyzing and graphing data. All cell culture data are expressed as mean ± SD of three independent experiments performed, at least and stated if differently, in duplicate. For the Presto-Blue viability assay, data were normalized for the untreated control (100% viability) and the TC_50_ was determined using the nonlinear fit “log(inhibitor) vs. normalized response with variable slope” with Graphpad prism from the viability % values. Statistical analysis was performed using one-way ANOVA analysis followed by Dunnett’s multiple comparison test. Gene expression and caspase activation was compared with control group using one-way ANOVA and Dunnett test, p < 0.05 was considered as significant.

## Supplementary information

Supplementary file

## Data Availability

Crystallographic data ‘CCDC 1846149’ is deposited at the Cambridge Crystallographic Data Centre and can be accessed via www.ccdc.cam.ac.uk/data_request/cif.

## References

[1] Jin C, Reed JC (2002). Yeast and apoptosis. Nat Rev Mol Cell Biol.

[2] Florea AM, Busselberg D (2011). Cisplatin as an anti-tumor drug: cellular mechanisms of activity, drug resistance and induced side effects. Cancers (Basel).

[3] Rybak LP, Mukherjea D, Jajoo S, Ramkumar V (2009). Cisplatin ototoxicity and protection: clinical and experimental studies. Tohoku J Exp Med.

[4] Montazerozohori M, Joohari S, Musavi SA (2009). Synthesis and spectroscopic studies of some cadmium(II) and mercury(II) complexes of an asymmetrical bidentate Schiff base ligand. Spectrochim Acta A Mol Biomol Spectrosc.

[5] Allardyce CS, Dyson PJ (2016). Metal-based drugs that break the rules. Dalton Trans.

[6] Gasser G, Ott I, Metzler-Nolte N (2011). Organometallic anticancer compounds. J Med Chem.

[7] Wani WA (2016). Recent advances in iron complexes as potential anticancer agents. New Journal of Chemistry.

[8] Ott I (2009). On the medicinal chemistry of gold complexes as anticancer drugs. Coordination Chemistry Reviews.

[9] Messner B, Turkcan A, Ploner C, Laufer G, Bernhard D (2016). Cadmium overkill: autophagy, apoptosis and necrosis signalling in endothelial cells exposed to cadmium. Cell Mol Life Sci.

[10] Yang H, Shu Y (2015). Cadmium transporters in the kidney and cadmium-induced nephrotoxicity. Int J Mol Sci.

[11] Abdel-Rahman LH, Abu-Dief AM, El-Khatib RM, Abdel-Fatah SM (2016). Some new nano-sized Fe(II), Cd(II) and Zn(II) Schiff base complexes as precursor for metal oxides: Sonochemical synthesis, characterization, DNA interaction, *in vitro* antimicrobial and anticancer activities. Bioorg Chem.

[12] Andelkovic K (2017). Synthesis, characterization and crystal structures of two pentagonal-bipyramidal Fe(III) complexes with dihydrazone of 2,6-diacetylpyridine and Girard’s T reagent. Anticancer properties of various metal complexes of the same ligand. J Inorg Biochem.

[13] Jazestani M, Chiniforoshan H, Tabrizi L, McArdle P (2017). Synthesis and crystal structures of cobalt(II), cadmium(II), and zinc(II) complexes of 4-nitro phenylcyanamide: enhancing the biological properties through bound to human serum albumin. J Biomol Struct Dyn.

[14] Hopa C, Yildirim H, Kara H, Kurtaran R, Alkan M (2014). Synthesis, characterization and anti-proliferative activity of Cd(II) complexes with NNN type pyrazole-based ligand and pseudohalide ligands as coligand. Spectrochim Acta A Mol Biomol Spectrosc.

[15] Yousef TA, Ahmed SF, El-Gammal OA, Abu El-Reash GM (2015). Structural, spectral, thermal and biological studies on (Z)-N-benzoyl-N′-(2-oxo-2-(phenylamino)acetyl)carbamohydrazonothioic acid (H(2)PABT) and its Cd(II), Hg(II), Zn(II) and U(VI)O(2)(2)(+) complexes. Spectrochim Acta A Mol Biomol Spectrosc.

[16] Perez JM (2001). DNA interstrand cross-linking efficiency and cytotoxic activity of novel cadmium(II)-thiocarbodiazone complexes. Chembiochem.

[17] Zec M (2014). Novel selenosemicarbazone metal complexes exert anti-tumor effect via alternative, caspase-independent necroptotic cell death. Med Chem.

[18] Flora SJ, Pachauri V (2010). Chelation in metal intoxication. Int J Environ Res Public Health.

[19] Zhou X (2015). Cadmium-coordinated supramolecule suppresses tumor growth of T-cell leukemia in mice. Cancer Sci.

[20] Kim JJ, Kim YS, Kumar V (2019). Heavy metal toxicity: An update of chelating therapeutic strategies. J Trace Elem Med Biol.

[21] Serda M (2014). Exploring the anti-cancer activity of novel thiosemicarbazones generated through the combination of retro-fragments: dissection of critical structure-activity relationships. PLoS One.

[22] Huls M, Russel FG, Masereeuw R (2009). The role of ATP binding cassette transporters in tissue defense and organ regeneration. J Pharmacol Exp Ther.

[23] Jansen J (2014). A morphological and functional comparison of proximal tubule cell lines established from human urine and kidney tissue. Exp Cell Res.

[24] Nieskens TT (2016). A Human Renal Proximal Tubule Cell Line with Stable Organic Anion Transporter 1 and 3 Expression Predictive for Antiviral-Induced Toxicity. AAPS J.

[25] Wilmer MJ (2010). Novel conditionally immortalized human proximal tubule cell line expressing functional influx and efflux transporters. Cell Tissue Res.

[26] Caetano-Pinto P (2016). Fluorescence-Based Transport Assays Revisited in a Human Renal Proximal Tubule Cell Line. Mol Pharm.

[27] Bhaskar V, Mohite P (2010). Synthesis, characterization and evaluation of anticancer activity of some tetrazole derivatives. Journal of optoelectronics and biomedical materials.

[28] Gardner SN (2002). Cell cycle phase-specific chemotherapy: Computation methods for guiding treatment. Cell Cycle.

[29] Dasari S, Tchounwou PB (2014). Cisplatin in cancer therapy: molecular mechanisms of action. European journal of pharmacology.

[30] Cande, C., Vahsen, N., Garrido, C. & Kroemer, G. (Nature Publishing Group, 2004). 10.1038/sj.cdd.4401400 15017385

[31] Wlodkowic, D., Skommer, J. & Darzynkiewicz, Z. In *Apoptosis* 19–32 (Springer, 2009). 10.1007/978-1-60327-017-5_2 PMC3863590 19609746

[32] Eom HJ, Choi J (2010). p38 MAPK activation, DNA damage, cell cycle arrest and apoptosis as mechanisms of toxicity of silver nanoparticles in Jurkat T cells. Environ Sci Technol.

[33] Soo JY, Jansen J, Masereeuw R, Little MH (2018). Advances in predictive *in vitro* models of drug-induced nephrotoxicity. Nat Rev Nephrol.

[34] Okuno A (1998). Troglitazone increases the number of small adipocytes without the change of white adipose tissue mass in obese Zucker rats. J Clin Invest.

[35] Denmeade, S. R., Lin, X. S., Tombal, B. & Isaacs, J. T. J. T. P. Inhibition of caspase activity does not prevent the signaling phase of apoptosis in prostate cancer cells. *Prostate* **39**, 269–279 (1999). 10.1002/(sici)1097-0045(19990601)39:4<269::aid-pros7>3.0.co;2-f 10344216

[36] Rani A, Kumar A, Lal A, Pant M (2014). Cellular mechanisms of cadmium-induced toxicity: a review. Int J Environ Health Res.

[37] Amendola V, Boiocchi M, Fabbrizzi L, Mosca L (2008). Metal-controlled anion-binding tendencies of the thiourea unit of thiosemicarbazones. Chemistry.

[38] Lessa JA (2011). Antimony(III) complexes with pyridine-derived thiosemicarbazones: Structural studies and investigation on the antitrypanosomal activity. Polyhedron.

