# Supplementary material for: In vitro nephrotoxicity and anticancer potency of newly synthesized cadmium complexes
Source: Sci Rep. 2019 Oct 11;9:14686. doi: 10.1038/s41598-019-51109-9 (PMC6789105; doi:10.1038/s41598-019-51109-9)
Supplement: Supplementary file 1 — Supplementary file [file 41598_2019_51109_MOESM1_ESM.pdf]

Supplementary data

In *vitro* nephrotoxicity and anticancer potency of newly synthesized cadmium complexes

Selda Abyar[a], Ali Akbar Khandar*[a], Roya Salehi*[b], Seyed Abolfazl Hosseini-Yazdi [a], Effat Alizadeh[b], Mehrdad Mahkam[c], Amer Jamalpoor[d], Jonathan M. White[e], Motahhareh Shojaei[a], O. Aizpurua-Olaizola[f], Rosalinde Masereeuw[d] and Manoe J. Janssen*[d]

[a] Department of Inorganic Chemistry, Faculty of Chemistry, University of Tabriz, Tabriz 5166614766, Iran

[b] Drug Applied Research Center and Department of Medical Nanotechnology, Faculty of Advanced Medical Science, Tabriz University of Medical Sciences, Tabriz, 51656-65811, Iran

[c] Chemistry Department, Faculty of Science, Azerbaijan Shahid Madani University, Tabriz, 5375171379, Iran

[d] Division of pharmacology, Utrecht Institute for Pharmaceutical Sciences, Utrecht University, Universiteitsweg 99, 3584 CG Utrecht, Netherlands

[e] School of Chemistry and BIO-21 Institute, University of Melbourne, Parkville, Vic. 3010, Australia

[f] Department of Chemical Biology and Drug Discovery, Utrecht Institute for Pharmaceutical Sciences, Utrecht University, Universiteitsweg 99, Utrecht, Netherlands

The grouping of gels/blots cropped from different parts of the same gel and cis in the figures is abbreviation for cisplatin

Caspase 7

A549 MCF-7

(ctrl) {2} {1} (ctrl) cis {2} {1}

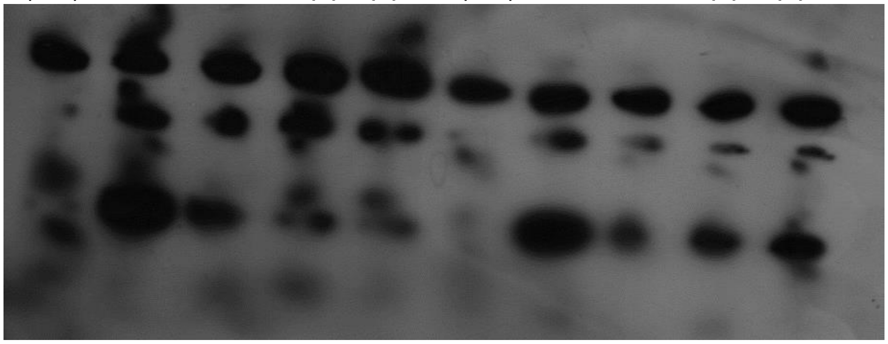

B-actin for caspase 7

A549 MCF-7

(ctrl) {2} {1} (ctrl) cis {2} {1}

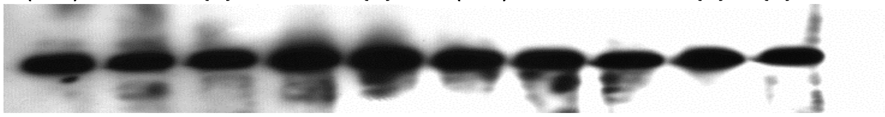

Caspase 8

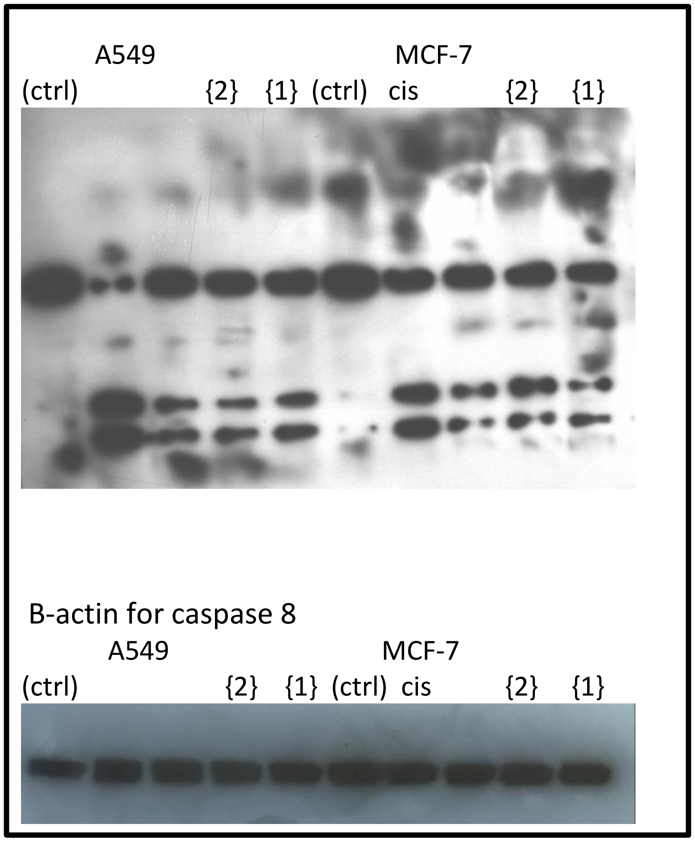
A549
MCF-7
(ctrl)
{2}
{1}
(ctrl)
cis
{2}
{1}
B-actin for caspase 8
A549
MCF-7
(ctrl)
{2}
{1}
(ctrl)
cis
{2}
{1}
